# Differentiation of Atrial Fibrillation and Atrial Fibrillation-Associated Ischemic Stroke Based on Serum Exosome miRNA-Seq

**DOI:** 10.1159/000529043

**Published:** 2023-02-09

**Authors:** Yun Xie, Lili Zhou, Meiyu Yan, Wei Zhao, Zhaoyang Hu

**Affiliations:** ^a^Department of Cardiology, Shanghai Putuo District People's Hospital, School of Medicine, Tongji University, Shanghai, China; ^b^Department of Clinical Medicine, Shanghai University of Medicine and Health Sciences, Shanghai, China; ^c^Department of Internal Neurology, Shanghai Putuo District People's Hospital, School of Medicine, Tongji University, Shanghai, China; ^d^Fun-med Pharmaceutical Technology (Shanghai) Co., Ltd., Shanghai, China

**Keywords:** Atrial fibrillation, Atrial fibrillation-associated ischemic stroke, Exosomes, miRNA-seq

## Abstract

**Introduction:**

Atrial fibrillation (AF) is the most common cardiac arrhythmia in the general population, and stroke is the most severe complication of AF. Exosomal miRNAs have been reported to be candidates as biomarkers for cardiovascular diseases, including AF and stroke. This study aimed to identify differentially expressed miRNAs (DEMs) in serum exosomes of AF and AF-associated ischemic stroke (AF-IS) patients and evaluate their potential in distinguishing AF and AF-IS patients.

**Methods:**

Serum exosomes were isolated from 8 healthy individuals with sinus rhythm (SR controls), 8 AF patients, and 8 AF-IS patients. miRNA-seq was performed to identify DEMs, and qRT-PCR analysis was performed to confirm the sequencing results. A support vector machine (SVM) model was developed using Python to distinguish AF and AF-IS patients.

**Results:**

68 and 86 DEMs were identified in serum exosomes of AF patients compared to AF-IS patients and SR controls, respectively. Levels of miR-641 and miR-30e-5p were found significantly higher in AF-IS patients. The SVM model achieved an accuracy of 100%, with an area under curve of 1.

**Conclusions:**

The results indicated that miRNA expression profiles of serum exosomes in AF patients were distinct from those in AF-IS patients, and based on the distinction, AF and AF-IS patients can be distinguished.

## Introduction

Atrial fibrillation (AF) is the most common arrhythmia. In China, the weighted AF prevalence was 1.8%, equating to affecting about 7.9 million people [1]. Stroke is the most mortal complication of AF, and its prevention is hence considered essential for AF management [2]. Currently, CHA_2_DS_2_-VASc, a clinical risk score, is still the most commonly used guideline to assess an individual's need for stroke prevention [3, 4, 5]. Although the CHA_2_DS_2_-VASc score is simple and costless, its performance in predicting the risk of stroke in real-world cohorts has been demonstrated to be modest [6]. This limitation is due to the score accounting for only several clinical risk factors (age, hypertension, diabetes, etc.). Thus, tools that consider more factors and features which can comprehensively reflect the condition of AF patients might aid in better stroke risk determination in these patients.

aaaaWith the widespread application of high-throughput sequencing, many more genetic characteristics of diseases can be accessed than ever. Recent studies have shown that exosomal miRNAs are potential biomarkers of cardiac diseases [7]. Wang et al. [8] reported 39 differentially expressed miRNAs (DEMs) in plasma exosomes of AF and sinus rhythm (SR controls) patients. Mun et al. [9] reported 5 miRNAs (miRNA-103a, −107, −320d, −486, and let7b) with significantly higher levels in serum exosomes of AF patients. Ji et al. [10] reported that serum exosomal miRNA-9 and miRNA-124 were expressed at significantly higher levels in stroke patients. Overall, these results suggest that exosomal miRNAs might be altered in AF and stroke patients. Among the 39 DEMs Wang et al. reported, miR-223 was also reported by Chen et al. [11] as a potential biomarker for ischemic stroke diagnosis. Considering AF is associated with the risk of stroke, it is not surprising to find DEMs associated with stroke appearance in AF patients. Therefore, exosomal miRNAs might act as promising biomarkers to identify AF patients with a higher risk of stroke. Studies focusing on AF and AF-associated ischemic stroke (AF-IS) patients might be instructive.

In this study, we started from identification of DEMs in serum exosomes of AF and AF-IS patients to establishment of a support vector machine (SVM) model through machine learning, aiming to evaluate the potential of serum exosomal miRNAs in distinguishing AF and AF-IS patients. Also, another differential expression analysis was performed to identify DEMs in healthy individuals with sinus rhythm and AF patients.

## Method

### Clinical Sample Collection

Totally, 8 healthy individuals, 8 AF patients, and 8 AF-IS patients were recruited from Putuo District People's Hospital, Shanghai, China. All participants included in the study were more than 18 years old. The control group comprised patients without AF and SR controls.

The inclusion criteria for the AF group were as follows: (1) patients with AF (including paroxysmal AF and persistent AF); (2) male patients with CHA_2_DS_2_-VASc score ≥2; (3) female patients with CHA_2_DS_2_-VASc score ≥3. The diagnosis of AF was made on the basis of the 2020 ESC Guidelines on the Diagnosis and Management of Atrial Fibrillation [12]. The inclusion criteria for the AF-IS group were as follows: (1) patients with AF-IS (including paroxysmal AF and persistent AF); (2) male patients with CHA_2_DS_2_-VASc score ≥2; (3) female patients with CHA_2_DS_2_-VASc score ≥3. The diagnosis of acute ischemic stroke was made on basis of CT/MRI examination.

The exclusion criteria were as follows: (1) patients with valvular heart disease; (2) patients received surgery within 3 months; (3) patients received anticoagulant therapy within 1 month; (4) patients with severe renal insufficiency; (5) patients with immune disease; (6) patients with infection; (7) patients with a history of tumor; (8) patients with a history of hematopathy and active bleeding; (9) patients with a history of thrombosis.

The study was approved by the Ethical Committee of the Putuo People's Hospital. Written informed consent was obtained from all participants.

### Exosome Isolation Characterization

Exosomes were isolated from the serum of healthy individuals, AF, and AF-IS patients. Briefly, 0.2-fold the volume of Total Exosome Isolation Reagent was added to the serum, followed by an incubation step at 4°C for 30 min. After that, the mix was centrifuged at 10,000 × *g* for 2 min, and the supernatant was removed. Another centrifugation step at 10,000 × *g* for 1 min was subsequently performed to further remove the residual supernatant. Finally, 110 µL of 1 × PBS was added to the precipitate, and the mix was incubated for 3 min, followed by a shaking step to resuspend exosomes.

For transmission electron microscopy (TEM) of the exosomes, 10 µL of the sample was dropped onto a copper grid and left to precipitate for 1 min. After blotting the grid with filter paper, the grid surface was touched with 10 µL of 2% phosphotungstic acid solution for 1 min. The excess phosphotungstic acid was removed by filter paper blotting, and the microscopy images were captured by a HT-7800 transmission electron microscope (HITACHI, Japan) operating at 80 kV. The mean size and particle concentration of the exosomes were determined by nanoparticle tracking analysis (NTA) using the Flow NanoAnalyzer (NanoFCM, China) according to the manufacturer's instructions.

Western blot analyses were performed to identify exosomal markers in exosomes. Briefly, proteins were resolved by SDS-PAGE and electro-transferred to PADF membranes (Millipore, MA, USA). The membranes were then blocked in 5% skim milk in Tris-buffered saline containing 0.1% Tween-20 (TBST) for 1 h at room temperature and subsequently incubated overnight at 4°C with anti-CD63 (1:1000; Abcam), anti-CD81 (1:1000; Abcam), anti-TSG101 (1:1000; Abcam), and anti-Calnexin (1:1000; Abcam) antibodies. After that, the membranes were washed 3 times with 20 mL TBST, followed by incubation with respective horseradish peroxidase-conjugated secondary antibodies (1:5000; Abcam) in 5% skim milk in TBST for 1 h at room temperature. After that, the washing step above was repeated. Immunoreactive bands were visualized with an ECL Detection Kit (Millipore, USA), and images were captured by a ChemiDoc Image Analyzer (BioRad, USA).

### RNA Extraction

700 µL of QIAzol was added to the resuspended exosomes, followed by a shaking step for 1 min, a short centrifugation step, and an incubation step for 5 min at room temperature. 140 µL of chloroform/isoamyl alcohol (24:1) was then added to the incubated mix, followed by a strong shaking step and another incubation step for 3 min at room temperature. After that, the mix was centrifuged at 12,000 × *g* for 8 min, and the supernatant was transferred to a new 2.0 mL tube, to which absolute ethyl alcohol twice the volume of the supernatant was added. The mix was then added to a RNeasy MinElute spin column. After that, the column was washed once with 700 of µL RWT buffer and twice with 500 µL of RPE buffer, followed by a dry spin step at 12,000 × *g* for 2 min. The column was then transferred to a new collection tube. Finally, 20 µL of RNA-free water was added to the column, followed by an incubation step for 1 min at room temperature and a centrifugation step at 12,000 × *g* for 2 min. Total RNA was qualified and quantified using a Nano Drop and Agilent 2100 bioanalyzer (Thermo Fisher Scientific, USA).

### miRNA Library Construction and Sequencing

The library was prepared with 1 μg of total RNA for each sample. Total RNA was purified by electrophoretic separation on a 15% urea denaturing polyacrylamide gel electrophoresis gel and small RNA regions corresponding to the 18–30 nt bands in the marker lane (14–30 ssRNA Ladder Marker, TAKARA) were excised and recovered. Then the 18–30 nt small RNAs were ligated to adenylated 3′ adapters annealed to unique molecular identifiers, followed by the ligation of 5′adapters. The adapter-ligated small RNAs were subsequently transcribed into cDNA by SuperScript II Reverse Transcriptase (Invitrogen, USA) and then several rounds of PCR amplification with PCR Primer Cocktail and PCR Mix were performed to enrich the cDNA fragments. The PCR products were selected by agarose gel electrophoresis with target fragments of 110–130 bp, and then purified using QIAquick Gel Extraction Kit (QIAGEN, CA, USA). The library was quantified by qPCR (TaqMan Probe), and the distribution of the fragments size was analyzed using the Agilent 2100 Bioanalyzer. The final ligation PCR products were sequenced using the BGISEQ-500 platform (BGI-Shenzhen, China).

### qRT-PCR Analysis

Quantitative real-time PCR was performed in triplicate for each validation sample using the Thermo Fisher Scientific Real-Time PCR System (Thermo Fisher Scientific, USA). Each reaction mixture contained 10 μL of 2 × UltraSYBR Mixture, 2 μL of forward and reverse primers, and 8 μL of cDNA. Primers were designed using the publicly available software miRprimer and are shown in Table 1. The reaction conditions were 95°C (10 min), followed by 40 cycles of 95°C (15 s), 60°C (20 s), 72°C (25 s), and a final extension of 72°C (5 min). The expression levels of target genes were normalized to the expression of cel-miR-39 and calculated using the 2^−ΔΔCt^ method. When comparing different groups, the expression values were log transformed.

### Data Analysis and Differential Expression Analysis

The demographic and clinical characteristics were compared using Student's *t* test between 2 groups or among 3 groups via R. The raw sequencing data were processed using the following steps: remove low-quality reads; remove reads with 5 primer contaminants; remove reads without 3 primer; remove reads without insertion; remove reads with poly A; remove reads shorter than 18 nt. After filtering, the clean reads were aligned to miRBase version 20 (ftp://mirbase.org/pub/mirbase/CURRENT/genomes/hsa.gff3) with Bowtie2. The miRNA expression level was calculated using featureCounts. Differential expression analysis was performed using the bioconductor package DESeq2 based on the threshold of *p* < 0.05 and the absolute value of Log2fold change >1.

### Adonis Analysis

Permutational multivariate analysis (Adonis) was used to statistically test the effects of demographic and clinical factors on the expression profiles of DEMs via the R package vegan.

### Target Gene Prediction and Functional Enrichment Analyses

Bioconductor package miRNAtap was used to predict possible target genes of each miRNA in database DIANA (http://diana.imis.athena-innovation.gr). GO and KEGG pathway enrichment analyses were then performed to functionally cluster the genes via the R package clusterProfiler.

### Model Development

Python Scikit-learn library was used to develop an SVM model. A train_test_split method was used to split samples into training and testing sets according to a ratio of 40:60. The training set was used to train the model, and the testing set was used to test the performance of the model. After that, a Platt scaling method was used to further transform SVM outputs into probability distribution.

## Results

### Baseline Characteristics

The demographic and clinical characteristics of the participants are shown in Table 2. For sequencing samples, the mean age was 56.8 (37–64), 84.2 (66–91), and 81.4 (59–95) years old (*p* < 0.01) in the SR control, AF, and AF-IS groups of patients, respectively. Also, the difference in mean LA diameter among the 3 groups was significant (*p* < 0.01). However, there were no differences in the other characteristics among the 3 groups.

### Characterization of Serum Exosomes

**Fig. 1:**
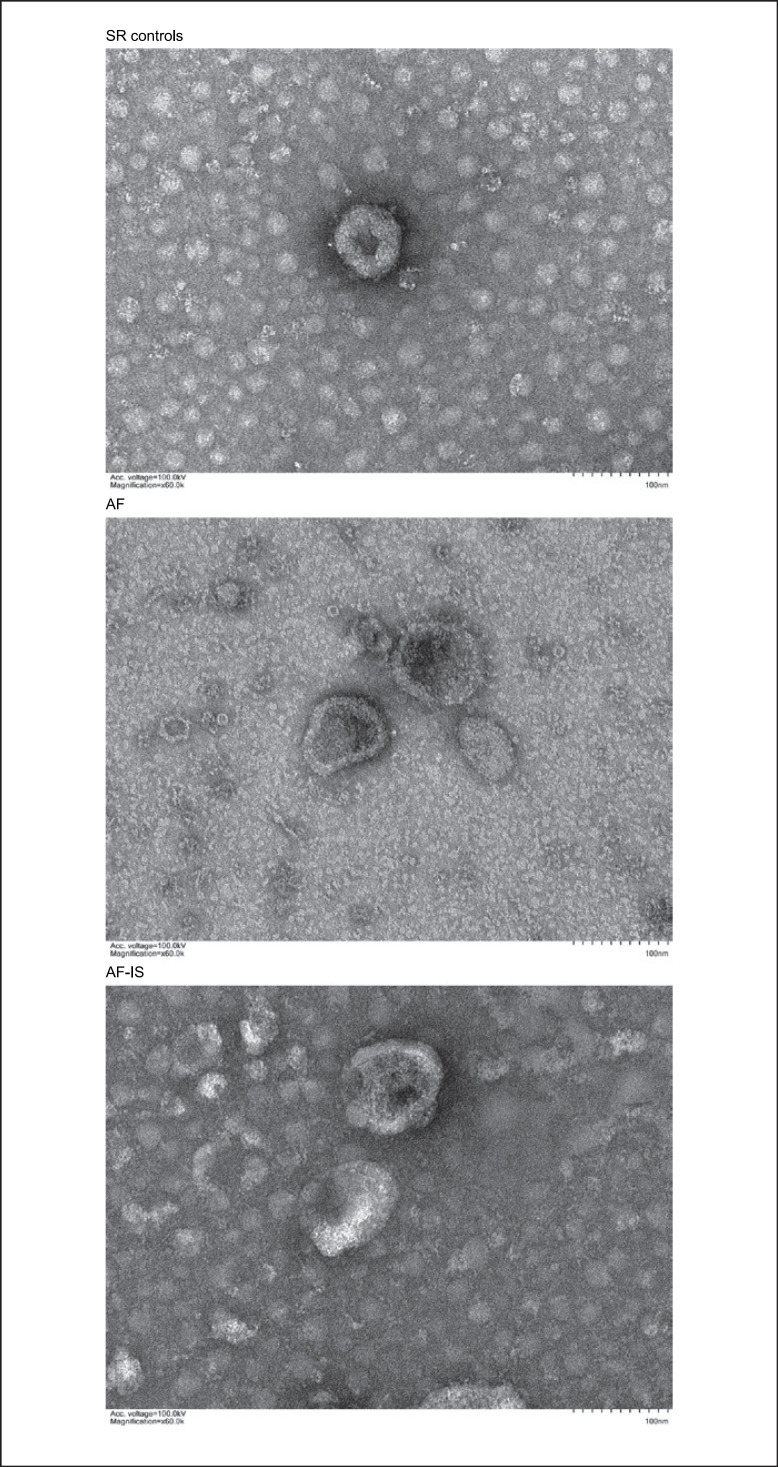
TEM results. TEM results show the size and morphology of the exosomes.

**Fig. 2:**
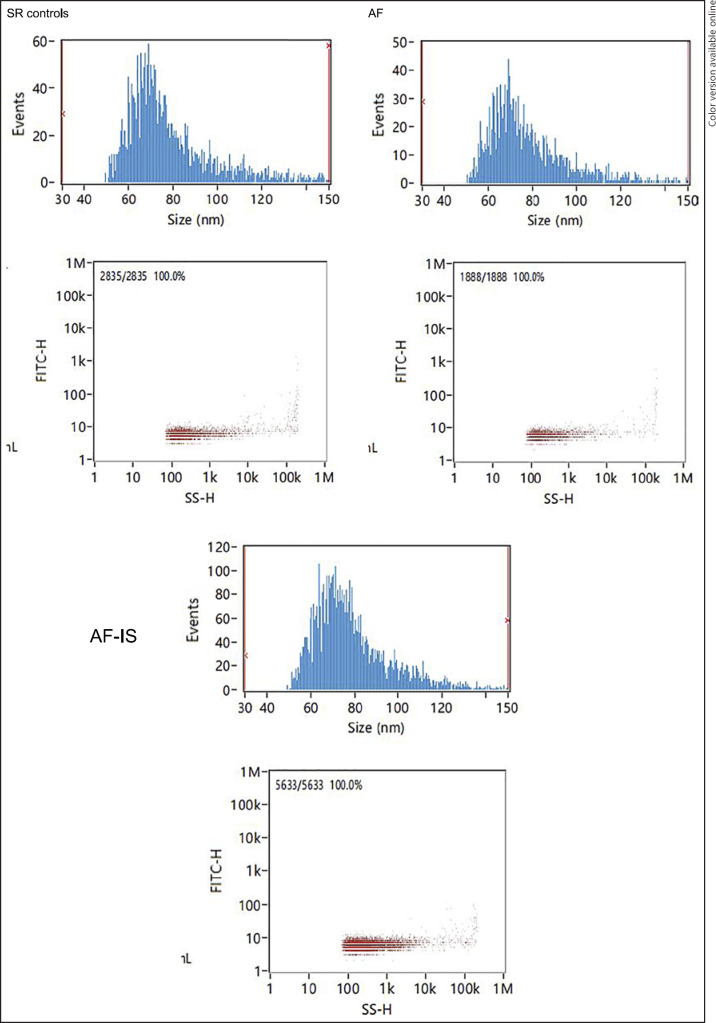
NTA results. NTA results show the size distribution and particle concentration of the exosomes.

**Fig. 3:**
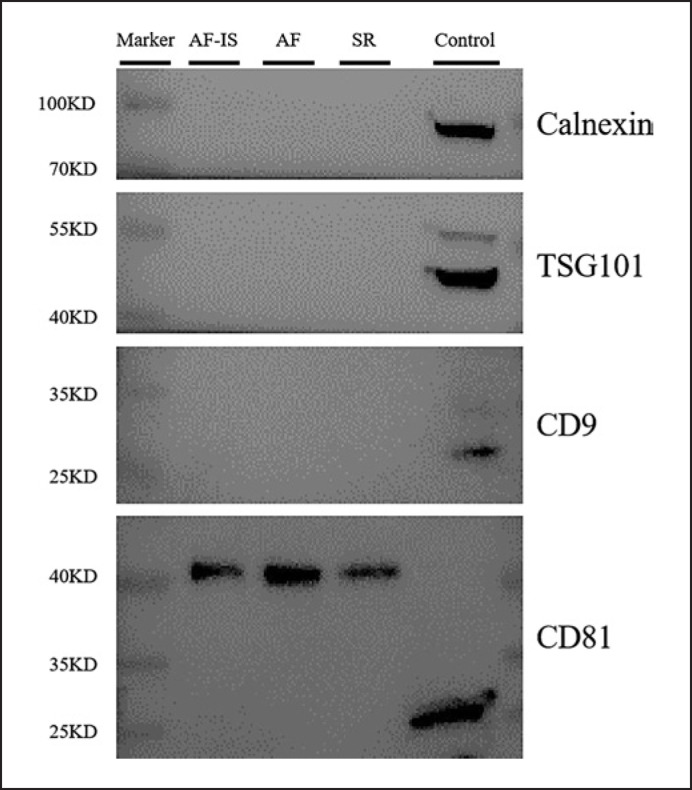
Western blot results. Western blot results confirmed the expression of the exosomal marker protein CD81 and Calnexin in the exosomes and control.

The identity and purity of the serum exosomes isolated from SR controls, AF, and AF-IS patients were characterized by TEM, NTA, and WB analysis. The size and morphology of the isolated exosomes was confirmed by TEM, in which exosome-like particle structures of 30–150 nm in size were identified as exosomes (Fig. 1). NTA demonstrated the size distribution and particle concentration of the exosomes, with an average particle diameter of 78.25 nm and concentration of 9.65E + 8 particles/mL for exosomes of the SR controls, 78.08 nm and 9.54E + 8 particles/mL for AF exosomes, and 78.63 and 2.90E + 9 particles/mL for AF-IS exosomes (Fig. 2). All exosomes were positive for the exosomal markers CD81 and negative for the exosomal marker Calnexin (Fig. 3).

### Analysis of miRNA Expression Profiles

Clean reads were annotated to a total of 2,657 miRNAs. 68 DEMs were identified in serum exosomes of AF patients compared to AF-IS patients, 31 with higher and 37 with lower expression (Table 3). Besides, 86 DEMs were identified in AF patients compared to SR controls.

### Effects of Clinical Phenotypes and Age on miRNA Expression Profiles

The Adonis results (online suppl. Table S1; for all online suppl. material, see www.karger.com/doi/10.1159/000529043) showed that in all 3 groups, the expression profiles of DEMs were significantly influenced by the clinical phenotypes (Adonis; *p* < 0.05) and not by age (Adonis; *p* > 0.05).

### Target Gene Prediction and Functional Enrichment Analyses

22,708 target genes were identified for the 31 increasingly expressed miRNAs, and 20,232 for the 37 decreasingly expressed miRNAs. GO analysis revealed that most target genes were enriched for biological processes such as histone modification, covalent chromatin modification, axonogenesis, and regulation of cell morphogenesis. KEGG pathway enrichment analysis demonstrated that the target genes of the 31 increasingly expressed miRNAs were mainly enriched in axon guidance, regulation of action cytoskeleton, Rap1 signaling pathway, and adherens junction; the target genes of the 37 decreasingly expressed miRNAs were mainly enriched in axon guidance, Wnt signaling pathway, signaling pathways regulating pluripotency of stem cells, and MAPK signaling pathway.

### qRT-PCR Validation

**Fig. 4:**
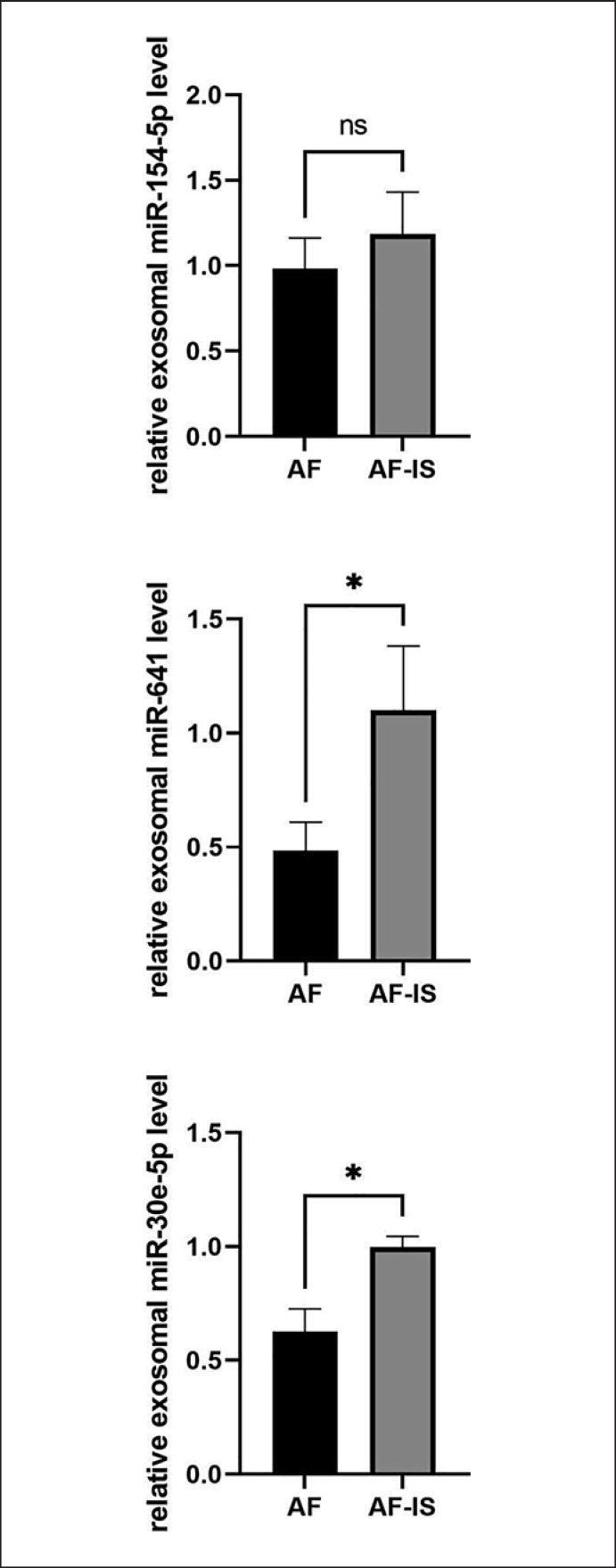
qRT-PCR results. The expression of exosomal miR-641 and miR-30e-5p was increased significantly in the AF-IS group (*n* = 3) compared to the AF group (*n* = 3). *p < 0.05.

3 miRNAs with reported roles in cardiovascular diseases were selected to confirm the miRNA-seq results between the AF and AF-IS patients. qRT-PCR analysis was performed on the 3 miRNAs that were significantly highly expressed in AF-IS patients: miR-154-5p, miR-641, and miR-30e-5p. Consistent with the miRNA-seq results, the expression levels of miR-641 (*p =* 0.0439) and miR-30e-5p (*p =* 0.0322) (Fig. 4) were significantly higher in AF-IS patients compared to AF patients. miR-154-5p (*p =* 0.3445) also showed an increased trend, but not significant.

### Exosome miRNA-Seq Allows for Classification of AF and AF-IS

The SVM model was trained with the training set (*n* = 4), and it employed the 68 DEMs as classifiers for sample classification. Subsequent testing was performed with the testing set (*n* = 6), yielding an accuracy of 100%, with an area under the curve of 1. Probability distribution of the testing samples is shown in Table 4. Among the 3 AF samples, sample AF_3 was observed to have much higher AF-IS probability (34.3% vs. 18.5% and 18.6%).

## Discussion

miRNAs, important regulators of mRNA expression, play vital roles in cell proliferation, differentiation, development, and death [13]. Abnormal expression of miRNAs has been reported in numerous diseases including cancer, neurological, and cardiovascular diseases [14, 15, 16].

Exosomes are 30–150 nm secreted membranous vesicles. These vesicles carry proteins, mRNA, miRNA, and noncoding RNAs, and play vital roles in intercellular communications and biologic functions [17, 18]. As exosome encapsulation protects exosomal miRNAs from degradation, exosomal miRNAs can provide more stable miRNA patterns to reflect pathological conditions [19, 20]. Exosomal miRNAs as biomarkers in AF and stroke have been reported [8, 9, 10, 11]. However, the use of exosomal miRNA to distinguish AF and AF-IS patients has not been seen.

In this study, for the first time, serum exosomal DEMs between AF and AF-IS patients were revealed. Based on them, an SVM model was developed and achieved high accuracy, sensitivity, and specificity in distinguishing AF and AF-IS patients. Also, important pathways between AF and AF-IS patients were identified. Previously, similar studies on AF usually stopped at revealing exosomal DEMs in AF patients. Wang et al. [8] identified 39 DEMs between AF and SR patients, and Mun et al. [9] identified 49 DEMs between AF and supraventricular tachycardia patients. Following their path, we also identified 86 DEMs in AF patients, and among them 83 were novel and 3 (miRNA-184, miRNA-4326, and miRNA-4507) were found consistently differentially expressed in their studies. Besides, our study further contributed to build a connection between AF and stroke at the molecular level. We not only reported several novel biomarkers between AF and AF-IS patients but also provided a new perspective on explaining why some DEMs were altered in AF patients. For example, miRNA-320b, miRNA-223-3p, and miRNA-3916, which were reported to be differentially expressed in Wang's study, also showed abnormal expression patterns between AF and AF-IS patients in our study. The same situation was also observed for miRNA-4758-5p and miRNA-4429 in Mun's study. Back then, the expression patterns of these DEMs might be only associated with atrial fibrillation, while now they can be further connected with increased risk of stroke.

The expression levels of exosomal miR-154-5p, miR-641, and miR-30e-5p were significantly higher in AF-IS patients compared to AF patients and showed consistent trends in sequencing and qRT-PCR results. miRNA-154-5p was reportedly overexpressed in exosomes derived from human amniotic fluid stem cells in a stroke in vitro model [21]. Previous studies have shown that miRNA-154-5p plays a role in neuroprotective mechanisms. More specifically, miR-154-5p is known to target Dickkopf-related protein 2 to result in the up-regulation of β-catenin and activation of classical Wnt signaling pathway, both of which were crucial for the maintenance of synaptic structures and neuronal survival [22, 23]. However, these findings made miRNA-154-5p a potential biomarker for prognosis of neuronal damage in AF-IS patients, but not for prediction of AF patients with stroke risks. miR-641 was reported to be significantly highly expressed in ischemic stroke, and demonstrated to regulate most target genes in a miRNA-target gene regulatory network [24]. Moreover, miR-641 expression was found decreased in ox-LDL-treated VSMCs. Overexpression of miR-641 repressed cell proliferation, migration, and invasion in ox-LDL-induced VSMCs, which suggested that miR-641 served as a suppressor in atherosclerosis progression [25]. miR-30e-5p was found increased in plasma exosomes of patients with atherosclerosis. Overexpression of miR-30e-5p represses cholesterol efflux [26]. However, miR-30e-5p was also reported to have anti-atherosclerosis effects by inhibiting proliferation and migration and promoting apoptosis in VSMCs [27]. Although these findings have linked miR-641 and miR-30e-5p to atherosclerosis and cardiovascular diseases, their functions were still not well known in AF and stroke, therefore, they may be of interest for further research.

The property of an SVM model is finding a hyperplane that separates two sample sets. In this study, the 68 DEMs together constructed a high-dimensional space, in which each sequencing sample has their own coordinates. Using the training set, we found a hyperplane that divided the AF and AF-IS samples exactly, which was also proved feasible for the testing set. 100% classification accuracy of the model suggested that expression patterns of the 68 miRNAs between AF patients and AF-IS patients were distinct. Based on that, further development of a risk prediction model was possible. For example, linear dimensionality reduction technique like principal component analysis (PCA) can be used to form clusters of AF and AF-IS patients by reducing the dimensions of the data. After that, the Mahalanobis distance (MD) can be used to measure the distance between new samples and the clusters, in other words, to assess the similarity of miRNA expression features presented by new samples and samples in the clusters, to enable stroke risk prediction in AF patients. Here, restricted to our model development capabilities, we simply employed the Platt scaling method to convert SVM outputs to probabilities. Interestingly, among the 3 AF samples with probability distributions, 1 AF sample with much higher AF-IS probability was observed.

### Limitations

The criteria we used for enrolling SR controls was patients without AF and with SR. When we initially designed the criteria, we failed to realize SR controls may have other diseases, such as hypertension, diabetes, or coronary diseases, which also have effects on the miRNA expression profiles. Although our sequencing samples eventually showed general consistency in comorbidity, which was probably due to these comorbidities being common in an advanced age cohort, the influence of these risk factors could be eliminated by enrolling patients with completely same comorbidities.

The SVM model was developed based on quite a small number of samples. Its performance would be more convincing if more samples had been included. Further model development is warranted to include significantly more AF and AF-IS patient samples. Although sample AF_3 was observed to have much higher AF-IS probability, follow-up records show that the patient has not had a stroke so far, therefore, preliminary conclusions about the potential of the model in predicting stroke risk in AF patients cannot be drawn. Further assessment of the model performance is planned to be combined with a prospective observational study.

## Conclusion

We identified 68 serum exosomal DEMs in AF patients compared to AF-IS patients, and 86 in AF patients compared to SR controls. Among the former, the expression levels of 2 miRNAs (miR-641 and miR-30e-5p) were found significantly increased. Based on the 68 DEMs, an SVM model was developed to distinguish AF and AF-IS patients, yielding an accuracy of 100%, with an area under the curve of 1. These results are significant to elicit further development of a stroke risk prediction model in AF patients.

## Statement of Ethics

The study was conducted according to the guidelines of the Declaration of Helsinki, and approved by the Ethical Committee of the Putuo People's Hospital (NO.202126). Written informed consent was obtained from all participants.

## Conflicts of Interest Statement

The authors declare no conflict of interest.

## Funding Sources

This work was funded by Science and Technology Innovation Project of Health System in Putuo District, Shanghai (NO. ptkwws202218).

## Author Contributions

Yun Xie designed the study and wrote the manuscript. Meiyu Yan and Wei Zhao collected blood samples. Zhaoyang Hu performed exosome miRNA-seq and was responsible for interpretation of sequencing data. Meiyu Yan performed bioinformatic analyses. Wei Zhao developed the SVM model. Lili Zhou performed Adonis analysis and helped with other statistical analyses. All authors have read and agreed to the published version of the manuscript.

## Data Availability Statement

All data generated or analyzed during this study are included in this article. Further inquiries can be directed to the corresponding author.

## Supplementary Material

Supplementary data

## Figures and Tables

**Table 1:** The primers used in quantitative real-time PCR

Primer name	Sequences
hsa-miR-154-5p RT	GTCGTATCCAGTGCAGGGTCCGAGGTATTCGCACTGGATACGACCGAAGG
hsa-miR-154-5p qRT F	TAGGTTATCCGTGTTG
hsa-miR-641 RT	GTCGTATCCAGTGCAGGGTCCGAGGTATTCGCACTGGATACGACGAGGTG
hsa-miR-641 qRT F	GCCGAGAAAGACATAGGATAGAGT
hsa-miR-30e-5p RT	GTCGTATCCAGTGCAGGGTCCGAGGTATTCGCACTGGATACGACCTTCCA
hsa-miR-30e-5p qRT F	TCGGCAGGTGTAAACATCCTTGAC
cel-miR-39 RT	GTCGTATCCAGTGCAGGGTCCGAGGTATTCGCACTGGATACGACCAAGCT
cel-miR-39 qRT F	AGCCCGTCACCTGGTGTAAATC

**Table 2:** Characteristics of participants

	Sequencing samples (miRNA-seq)				Validation samples (qRT-PCR)		
Parameter	SR controls (*n* = 5)	AF (*n* = 5)	AF-IS (*n* = 5)	*p*	SR controls (*n* = 3)	AF (*n* = 3)	AF-IS (*n* = 3)
Age, years	56.8 (37–64)	84.2 (66–91)	81.4 (59–95)	<0.01	57.3 (50–62)	67 (65–70)	84.3 (82–89)
Male	1 (20)	2 (40)	2 (40)	0.7828	0 (0)	0 (0)	0 (0)
BMI, kg/m^2^	23.54 (19.5–30.4)	26.14 (23.6–28.2)	24.54 (21.4–26.7)	0.4094	24.4 (22–27)	22.9 (22.4–23.4)	25.0 (18.2–32.4)
CHA2DS2-VASc score	/	4.6 (3–7)	5 (3–7)	0.7204	/	4 (3–6)	6 (4–8)
Hypertension	4 (80)	4 (80)	4 (80)	0.2509	2 (66.7)	3 (100)	2 (66.7)
Diabetes	0	1 (20)	1 (20)	0.4718	0	0	2 (66.7)
Other CAD	0	0	0	/	0	3 (100)	0
LA diameter, mm	35.2 (32–42)	44.6 (41–46)	38.4 (34–41)	<0.01	36 (36)	40.7 (30–49)	42.7 (40–44)
LVEF, % Types of AF	65.2 (64–68)	59 (50–65)	62.8 (61–65)	0.055	62.3 (62–63)	62 (61–63)	62.7 (60–64)
Paroxysmal	/	2 (40)	2 (40)	/	/	3 (100)	3 (100)
Persistent	/	3 (60)	3 (60)	/	/	0 (0)	0 (0)
Statin	0 (0)	4 (80)	2 (40)	/	0 (0)	2 (66.7)	2 (66.7)

Values are expressed as *n* (%) or mean (range).

**Table 3:** DEMs between AF and AF-IS patients

Gene name	Base mean	Log2FoldChange	LfcSE	Stat	*p* value	*p* adj
hsa-miR-1285-5p	36.99496594	8.003162991	1.81342231	4.41329245	0.0000102	0.002280551
hsa-miR-1224-5p	22.52105573	7.8540081	2.528048292	3.106747653	0.001891578	0.090795741
hsa-miR-513c-5p	18.73950455	7.589683021	2.024591336	3.748748148	0.000177719	0.017061066
hsa-miR-6764-5p	18.29638608	7.551470681	1.963337392	3.846241972	0.000119943	0.013433651
hsa-miR-205-5p	17.64528623	7.502712317	2.563951889	2.926229758	0.003430974	0.121348149
hsa-miR-579-3p	14.77721976	7.243535586	3.028309071	2.391940656	0.016759552	0.316281948
hsa-miR-6837-5p	14.43171558	7.211030784	3.028357402	2.381169006	0.017257792	0.316281948
hsa-miR-4796-3p	13.20568443	7.081195646	3.028561676	2.338138167	0.019380082	0.325585375
hsa-miR-4731-5p	11.17699346	6.839878769	3.028993743	2.258135654	0.023937203	0.355363822
hsa-miR-200b-3p	37.26596739	6.810461657	1.371611455	4.965299489	0.000000686	0.00023048
hsa-miR-4714-3p	9.7496734	6.645482851	3.029398273	2.193664303	0.028259552	0.374077526
hsa-miR-3127-5p	18.17871534	6.585663648	2.691128664	2.447175319	0.014398078	0.312113183
hsa-miR-1269b	8.509528392	6.450529714	2.090589012	3.085508283	0.002032046	0.091035645
hsa-miR-2355-3p	16.37799056	6.294102861	2.065173419	3.047735751	0.002305726	0.096840486
hsa-miR-362-3p	21.38116414	6.149130523	1.955031566	3.145284521	0.001659254	0.087325624
hsa-miR-4646-3p	9.67483576	6.014804945	2.853710808	2.107713552	0.035055774	0.385973074
hsa-miR-337-5p	17.73595967	5.544725053	2.209889384	2.509050948	0.012105602	0.28095725
hsa-miR-4479	43.04760452	5.336343973	2.262730582	2.358364719	0.018355649	0.316281948
hsa-miR-9-5p	342.7065962	3.367636534	1.253364572	2.686877074	0.007212349	0.211995166
hsa-miR-412-5p	93.10400219	3.014000875	1.440733211	2.091990976	0.036439323	0.385973074
hsa-miR-641	31.12438878	2.919518772	1.212313652	2.408220651	0.016030489	0.316281948
hsa-miR-338-5p	111.5104777	2.888562297	1.348699182	2.141739489	0.032214451	0.374077526
hsa-miR-5585-3p	74.88889577	2.741178199	1.301851679	2.105599466	0.035239159	0.385973074
hsa-miR-619-5p	314.4820175	2.229264104	0.694821072	3.208400254	0.001334756	0.087325624
hsa-miR-154-5p	61.76710852	2.184201512	0.860804837	2.537394561	0.0111681	0.277961611
hsa-miR-3187-3p	183.9701907	2.0103703	0.64024025	3.140024858	0.001689335	0.087325624
hsa-miR-30e-5p	100.7529283	1.670712308	0.833277942	2.004988041	0.044964326	0.413918175
hsa-miR-320b	3186.290904	1.554377076	0.763685142	2.035363779	0.041814283	0.395763355
hsa-miR-145-5p	593.8514433	1.538457893	0.613298987	2.508495733	0.012124643	0.28095725
hsa-miR-628-3p	222.5041417	1.369328177	0.632523776	2.164864357	0.030398074	0.374077526
hsa-miR-223-3p	13,179.07204	1.125516095	0.469074988	2.399437452	0.016420285	0.316281948
hsa-miR-125a-5p	12,428.28083	−1.010226011	0.485666185	−2.080083073	0.037517914	0.385973074
hsa-miR-139-5p	38,147.50562	−1.02690112	0.500529128	−2.051631088	0.040205529	0.385973074
hsa-miR-224-5p	1172.797023	−1.316574272	0.588559045	−2.2369451	0.025289928	0.355363822
hsa-miR-1-3p	658.0335212	−1.433387312	0.516976573	−2.772634946	0.005560446	0.17793427
hsa-miR-1260b	6914.138111	−1.433937442	0.498367822	−2.877267311	0.004011356	0.134781572
hsa-miR-1260a	2966.704821	−1.913618868	0.608613712	−3.144225688	0.00166527	0.087325624
hsa-miR-1306-5p	605.2425346	−2.019677651	0.524091063	−3.853676956	0.000116357	0.013433651
hsa-miR-3613-3p	19.8559386	−2.621352013	1.172590818	−2.235521524	0.02538313	0.355363822
hsa-miR-3916	44.85914494	−3.705401901	1.387471682	−2.670614433	0.007571256	0.211995166
hsa-miR-7155-5p	5.918681699	−3.903144097	1.649291824	−2.366557598	0.01795438	0.316281948
hsa-miR-548az-5p	8.020800449	−4.528603055	2.172085063	−2.084910546	0.037077421	0.385973074
hsa-miR-219a-5p	6.903742587	−5.164605511	1.926765744	−2.680453255	0.007352253	0.211995166
hsa-miR-3177-3p	17.14111599	−5.210593948	2.257993574	−2.307621248	0.021020212	0.338391609
hsa-miR-642a-3p	8.657890636	−5.511841374	1.707714841	−3.22761227	0.00124828	0.087325624
hsa-miR-642b-5p	3.8465287	−5.588181233	2.424052468	−2.305305395	0.021149476	0.338391609
hsa-miR-6754-3p	10.3009667	−5.730893846	2.553475116	−2.244350771	0.024809841	0.355363822
hsa-miR-3180-5p	11.04174702	−5.849359172	2.300617216	−2.542517344	0.011005714	0.277961611
hsa-miR-6884-3p	5.133960899	−6.006222214	2.993665352	−2.006310495	0.044823131	0.413918175
hsa-miR-1237-3p	5.721254549	−6.154570752	2.997911778	−2.052952591	0.040077176	0.385973074
hsa-miR-6743-5p	6.021125967	−6.227630337	3.032694484	−2.053497432	0.040024359	0.385973074
hsa-miR-4489	6.081198768	−6.246258274	3.032611216	−2.059696357	0.039427578	0.385973074
hsa-miR-202-3p	6.288172135	−6.291971651	3.032411354	−2.074907035	0.037995152	0.385973074
hsa-miR-3137	6.356517868	−6.305544009	3.032353218	−2.079422665	0.037578519	0.385973074
hsa-miR-516a-5p	7.103809692	−6.469618032	3.031691869	−2.133995904	0.032843116	0.374077526
hsa-miR-7112-3p	7.108254721	−6.470959811	3.031686764	−2.134442083	0.03280661	0.374077526
hsa-miR-5706	7.167987932	−6.483794726	3.031638165	−2.138709956	0.032459166	0.374077526
hsa-miR-643	7.22995744	−6.492346051	3.031606023	−2.141553355	0.032229441	0.374077526
hsa-miR-1908-3p	11.67242088	−6.59739211	2.793538339	−2.36166156	0.018193243	0.316281948
hsa-miR-6755-3p	7.858042046	−6.614346507	2.659459578	−2.487101726	0.012878857	0.28848639
hsa-miR-200a-5p	11.99871776	−6.642073206	2.197178861	−3.023000687	0.002502817	0.098934874
hsa-miR-4802-5p	8.458164574	−6.721852001	3.030810645	−2.217839644	0.026565768	0.364330532
hsa-miR-6504-5p	8.984951962	−6.811945484	2.566150346	−2.654538732	0.007941692	0.213472686
hsa-miR-5193	11.63465611	−7.184851143	3.029543443	−2.37159535	0.017711476	0.316281948
hsa-miR-6821-5p	12.28376448	−7.263452227	1.706681978	−4.255890857	0.0000208	0.003498067
hsa-miR-1299	13.35625096	−7.383046113	2.467157478	−2.992531356	0.002766742	0.103291718
hsa-miR-4433b-3p	19.08156477	−7.897470991	2.377603824	−3.321609307	0.000894999	0.075179931
hsa-miR-6850-5p	36.80767603	−8.264120029	1.229573374	−6.721127998	1.8E–11	1.21E–08

**Table 4:** Probability distribution of SVM outputs

Sample No.	AF probability	AF-IS probability
AF_3	0.65679423	0.34320577
AF-IS_4	0.18022012	0.81977988
AF_5	0.81503718	0.18496282
AF-IS_5	0.06644224	0.93355776
AF_2	0.81439552	0.18560448
AF-IS_2	0.11808278	0.88191722
