# Supplementary material for: Differentiation of Atrial Fibrillation and Atrial Fibrillation-Associated Ischemic Stroke Based on Serum Exosome miRNA-Seq
Source: Cardiology. 2023 Feb 9;148(2):150–60. doi: 10.1159/000529043 (PMC10273881; doi:10.1159/000529043)
Supplement: Supplementary file 1 — Supplementary data [file crd-0148-0150-s01.doc]

Table S1. Effects of clinical phenotypes and age on miRNA expression profiles

| Group | Age | Clinical phenotypes |
| --- | --- | --- |
| AF vs NM | 0.061 | 0.026 |
| AF-IS vs NM | 0.496 | 0.008 |
| AF-IS vs AF | 0.321 | 0.095 |
